# Detection of Soluble ST2 in Human Follicular Fluid and Luteinized Granulosa Cells

**DOI:** 10.1371/journal.pone.0074385

**Published:** 2013-09-10

**Authors:** Jennifer H. Southcombe, Nathalie Lédée, Sophie Perrier d’Hauterive, Karen Turner, Tim Child, James V. Snider, Christopher W. G. Redman, Ian L. Sargent, Ingrid Granne

**Affiliations:** 1 Nuffield Department of Obstetrics and Gynaecology, University of Oxford, Oxford, United Kingdom; 2 Université Paris Diderot and INSERM U976, Paris, France; 3 Medicine of Experimental Reproduction and Centre de Procréation Médicalement Assistée, University of Liège, Liège, Belgium; 4 Oxford Fertility Unit, University of Oxford, Oxford, United Kingdom; 5 Critical Diagnostics, San Diego, California, United States of America; University of Oslo, Norway

## Abstract

Follicular fluid (FF) contains various cytokines that are involved with folliculogenesis, some of which have been shown to be associated with oocyte quality and the implantation potential of a resulting embryo. Several IL-1 family members have previously been identified in FF. This study investigates a newly identified member of the family, IL-33, and its receptor ST2, comparing values to those of FF Granulocyte-Colony Stimulating Factor (G-CSF) – a known predictor of Assisted Reproductive Technology (ART) success. FF was collected from patients undergoing *in vitro* fertilisation/intra-cytoplasmic sperm injection (IVF/ICSI) at oocyte retrieval to analyse IL-33 and sST2 expression in human follicles. sST2, but not IL-33, is highly increased in the FF compared to plasma levels (up to 7.9-fold), with higher levels in larger follicles (p<0.05). Furthermore, we identify that human luteinised granulosa cells are one possible source of the FF sST2, as these cells express and secrete sST2 when cultured *ex vivo*. FF associated with oocytes which when fertilised develop into good quality embryos have higher sST2 levels than those which are graded average (p<0.01). These embryos were transferred to the patient and levels of FF sST2 compared between successful and unsuccessful ICSI cycles. However unlike G-CSF, sST2 levels cannot be used to predict cycle outcome.

## Introduction

Follicular fluid (FF) contains molecules that either enter into the follicle from the plasma or are secreted from resident follicular cells, such as thecal and granulosa cells or possibly from the oocyte itself. Along with hormones and growth factors a number of cytokines have been identified within the FF [1]. Cytokines are immune cell mediators with numerous functions; for example they are known to stimulate or inhibit cell growth, trigger cell migration and help initiate an inflammatory response. Within the ovary, cytokines have been implicated in the processes of follicle growth, steroidogenesis, ovulation and luteinisation [2]. Reproductive disorders have been associated with dysregulation of cytokine levels, and in recent years FF levels of various cytokines have been related to oocyte quality and IVF/ICSI cycle success rate [3]. Granulocyte-Colony Stimulating factor (G-CSF) is a proven non-invasive FF biomarker of successful implantation [4]; a recent retrospective study suggested that measuring FF G-CSF levels may increase on-going pregnancy rates by up to 37.5% in fresh embryo transfers by improving embryo selection. However the availability of additional biomarkers would improve predictive capabilities.

The interleukin-1 (IL-1) cytokine family is important for female reproductive success [5], [6]. IL-1 was first identified as a T-lymphocyte activating factor [7], and two IL-1 genes were identified, now called IL-1α and IL-1β [8]. IL-1 is present in FF [9], and IL-1β is lower in FFs from women with successful ICSI attempts [10]. IL-1 receptor antagonist (IL-1ra) is another member of the IL-1 family, which blocks binding of IL-1α and IL-1β to their receptor IL-1R preventing their function. While IL-1ra has not been described in human FFs IL-1ra mRNA is detectable in human granulosa and cumulus cells [11]. The next cytokine to be identified in the IL-1 family was IL-18. FF IL-18 is thought to be important for folliculogenesis [12] as levels correlate with the number of oocytes retrieved in IVF cycles, in addition FF IL-18 levels are low in patients with unexplained infertility compared to patients with tubal factor infertility [3].

IL-33 is a newly described member of the IL-1 cytokine family. It has dual functions, acting as both a nuclear transcription factor and a cytokine [13]. In addition, secretion upon necrotic cell death triggers strong pro-inflammatory responses and it is classified as an ‘alarmin’ [14]. IL-33 binds to its cellular receptor ST2L, which is expressed by a number of cell types, particularly cells of the immune system [15], [16] triggering activation of signalling pathways which converge upon NFκB activation and typically type 2 cytokine immune responses (e.g.: IL-4 and TNFα) [17], [18]. Soluble ST2 (sST2) is a splice variant form of the ST2 gene which lacks the transmembrane and intracellular regions, and acts as a decoy receptor preventing IL-33 functions. In addition, sST2 may have other roles in regulation of cell growth, metastasis [19] and has direct anti-inflammatory properties through binding to monocytes (to an unknown receptor) [20].

Increased levels of sST2 in the maternal plasma have recently been associated with the reproductive disorder pre-eclampsia [21]. Both IL-33 and ST2 are expressed by trophoblast cells in the third trimester of pregnancy [21], amnion mesenchymal cells [22] and decidualised endometrial stromal cells [23]. Dysregulation of the IL-33/ST2 axis in the endometrium occurs in women with recurrent pregnancy loss [23]. As yet these molecules have not been investigated in the human ovary. We sought to investigate the levels of IL-33 and sST2 in human FFs derived from women undergoing IVF cycles. We found that sST2, but not IL-33, is greatly enriched in the FFs. Furthermore, FF sST2 levels were highly variable in different follicles from the same patient, and between patients. Luteinized granulosa cells were found to produce sST2; they also expressed IL-33 but did not constitutively secrete it. We further analysed fluids from follicles from ICSI cycles with known oocyte outcome. FF G-CSF levels are an established predictor of pregnancy success which aids embryo selection [4], therefore G-CSF levels were determined in parallel. Higher levels of sST2 were detectable in FF from oocytes which formed good quality compared to average quality embryos (p<0.01). sST2 is not an overall predictor of pregnancy success, however low levels of follicular sST2 were associated with good quality day 3 embryos which tended towards pregnancy success. This is in contrast to G-CSF which, as previously shown, is increased in the follicles of oocytes which are most likely to lead to a live birth.

## Methods

### Ethical Approval

Oxford Fertility Unit samples: these studies were approved by the Oxfordshire Research Ethics Committee C, approval number 08/H0606/94, and informed written consent was obtained from each participant. Liège University samples: This study was approved by the Local Ethics Committee of the Liege Medical School, in 2008. Written Information concerning the collection of individual FFs and their use in research was available to all patients attending the CAMP, Liege, Belgium. All patients provided informed verbal consent which was documented in their patient notes, as required and approved by the Liege Medical School ethics committee. At the time these samples were collected Belgium law (2008) did not require written consent for the use of biological samples, such as FF, which are a waste by-product of the IVF treatment cycle clinical procedure and are always discarded after the egg collection.

### Subject Recruitment

Oxford Fertility Unit samples: The patient characteristics of the 21 women recruited to the study are shown in table 1, and patients divided into those that became pregnant or did not become pregnant post ART. 10 patients had IVF and 11 patients ICSI treatments, infertility was primary for all patients, characterised as male (7), female (5), mixed (5) or unexplained (4) infertility. The mean (±1S.E.M.) patient age was 34.19(±0.64) years (table 1). The women had an average of 12.85(±1.46) oocytes retrieved, 68.12% fertilization rate. The clinical pregnancy rate was 33% and live birth rate 33%.

**Table 1:** Patient characteristics for women recruited to the Oxford Fertility Unit. Values are Mean (S.E.M).

	Age (years)	FSH (iU/L)	Collected oocytes (number)	Plasma sST2 (ng/ml)	FF sST2 (ng/ml)	Plasma IL-33 (pg/ml)	FF IL-33 (pg/ml)
All patients (n = 21)	34.19 (0.64)	6.63 (0.47)	12.85 (1.46)	29.76 (2.63)	76.62 (8.42)	18.95 (7.44)	17.60 (7.40)
Clinical Pregnancy (n = 7)	34.00 (0.73)	6.27 (0.47)	12.50 (2.51)	26.51 (2.71)	78.06 (14.35)	21.99 (13.56)	12.10 (9.47)
No Clinical Pregnancy (n = 14)	34.29 (0.89)	6.81 (0.66)	13.00 (1.78)	31.39 (3.64)	75.43 (38.85)	33.06 (8.84)	19.00 (9.98)

Liège University samples: All samples were from patients undergoing ICSI, 64 patients were recruited aged 33.19(±0.45) (table 2), of these patients 29 had male infertility, 13 had female infertility, 18 had mixed infertility and 4 were unexplained. Infertility was primary for 45 couples and secondary for 19 couples. FFs were collected from 113 follicles where the oocyte developed into an embryo which was transferred with a known outcome. 25 of the embryos resulted in a live birth (22%). 26 embryos were frozen prior to transfer (2 frozen embryos resulted in live birth). When two embryos were transferred only pregnancies that resulted in twin births on no live birth were included.

**Table 2:** Patient characteristics for women recruited to Liège University. Values are Mean (S.E.M.).

	Age (years)	Collected oocytes	FF sST2 (ng/ml)	FF G-CSF (pg/ml)
All Patients	33.19 (0.45)	11.67 (0.48)	61.37 (2.99)	24.74 (1.18)
Embryo grade Good (n = 52)	32.94 (0.67)	12.46 (0.69)	67.65 (4.13)	28.41 (1.69)
Embryo grade Average (n = 28)	33.50 (0.97)	10.61 (0.87)	48.23 (3.25)	27.34 (2.54)
Embryo grade Poor (n = 38)	33.95 (0.73)	11.55 (0.57)	58.12 (3.69)	24.24 (1.15)
Clinical Pregnancy (n = 29)	32.79 (0.85)	12.48 (0.92)	56.63 (4.12)	32.56 (2.01)
No clinical pregnancy (n = 84)	33.32 (0.53)	11.67 (0.48)	61.37 (2.99)	24.74 (1.112)

### Treatment

Oxford Fertility Unit: All participants underwent a standard long protocol IVF cycle. Down regulation was achieved using 400 mcg nafarelin acetate (Syneral, Pfizer, USA) twice daily. Daily subcutaneous recombinant FSH (Gonal-F, Merck-Serono, Switzerland) was injected with a reduced dose of nafarelin acetate until the decision for oocyte collection was made. The response to stimulation was monitored with serial ultrasound scans and plasma estrogen levels. HCG (Ovitrelle, 6500i.u., Merck-Serono) was given when 3 follicles had reached 18 mm in size. Oocyte retrieval was planned for 36 hours after HCG.

Liège University Samples: Of the 64 patients included in the study all underwent ICSI and the following ovarian hyperstimulation protocols were used; Standard long protocol with a daily GnRH agonist = 44, short protocol with a daily GnRH agonist = 17, antagonist protocol = 2, and 1 patient had a natural cycle. The treatments were as previously described using the same criteria to analyse embryo morphology on day 3 [4].

### FF and Plasma Collection and Sample Processing

Oxford Fertility Unit: FFs were collected from women undergoing IVF/ICSI. Follicle size was estimated as small (<12 mm), medium (12–17 mm) or large (>18 mm) and individual FFs collected after identification of the oocyte. A blood sample was collected into an EDTA vacutainer (BD Biosciences, UK) and immediately centrifuged at 2000 g for 10 minutes. Plasma was stored at −80°C for subsequent analysis. FFs were centrifuged at 13,000×g for 5 minutes to remove cellular debris, and supernatants frozen at −80°C for subsequent analysis.

Liège University : FFs were collected as previously described [4].

### Detection of sST2 and IL-33 by ELISA and G-CSF by Luminex

sST2 was measured in plasma using the Presage® ST2 Assay (Critical Diagnostics, San Diego, USA) according to the manufacturer’s instructions. IL-33 was measured in plasma using a human IL-33 ELISA kit (BioLegend, San Diego, USA). For each assay both standards and samples were tested in duplicate.

G-CSF was analysed using a Luminex System (Luminex XMap Technology from BioRad) [4].

### Enrichment of Granulosa Cells

FFs from individual patients (n = 3) were pooled and supernatants strained through a 70 µm Falcon Cell Strainer (BD Biosciences, UK). This retained the sheets of granulosa cells but single cell blood contaminants passed through. Granulosa cells were harvested and washed ×2 in PBS by centrifugation at 200×g for 5 minutes. Cells were incubated with 0.1% hyaluronidase (Sigma Aldrich, UK) for 20 minutes at 37°C, then washed as previously described. To confirm enrichment of granulosa cells, and elimination of contaminating leukocytes, cells from before and after processing were stained with antibodies towards CD45 (the leukocyte common antigen, not expressed by granulosa cells}; flow cytometry revealed >98% purity. Briefly, cells were incubated with 1 µg/ml CD45-APC antibody (BD Bioscience, UK) for 20 minutes at 4°C, washed ×2 in PBS/2% FCS and analysed immediately by flow cytometry on a LSR-II flow cytometer (BD Biosciences, Oxford, UK). Data was analysed using FACS DIVA software (BD Biosciences, Oxford, UK). Figures were generated using FlowJo software (Tree Star Inc., USA).

### Detection of ST2 and IL-33 by Immunoblotting

Isolated granulosa cells from all of the follicles from an individual patient (n = 3) or a 0.25 cm^2^ section of placenta, as a positive control, were lysed using HEPES lysis solution supplemented with Complete Mini protease inhibitor cocktail (Roche, Germany). Cell lysate protein concentrations were determined using a BCA protein assay kit (Pierce, Thermo Scientific, Illinois, USA). Proteins (20 µg) were resolved by 10% Bis-Tris Novex SDS-PAGE (Invitrogen, Paisley, UK) and transferred to PVDF membrane. Membranes were incubated for 1 hour in blocking buffer (PBS with 5% BLOTTO (Santa Cruz, California, USA) and 0.1% Tween) at room temperature. The membranes were then incubated overnight with the appropriate primary antibody at 4°C. Primary antibodies used were goat anti-ST2 (0.5 µg/ml) (R&D Systems, Minneapolis, USA), murine anti-IL-33 (0.1 µg/ml) clone Nessy-1 (Enzo Life Sciences, New York, USA) or murine anti-β-actin (0.155 µg/ml) (Abcam, Massachusetts, USA). Reactions were visualised by incubating the membranes with the appropriate anti-mouse or anti-goat secondary antibody conjugated to horseradish peroxidase (Dako, Denmark) for one hour and detected using an enhanced chemiluminescence system (Pierce, Thermo Scientific, Illinois, USA).

### Luteinized Granulosa Cell Culture

Isolated granulosa cells were re-suspended at a concentration of 10^6^/ml in growth medium (HAM F10 (Sigma, UK) containing 10% Human Serum (Sera Laboratories International, West Sussex, UK), 1% penicillin-streptomycin (50 IU/ml and 50 µg/ml), 1% glutamine) and cultured for 72 hours. At 24, 48 and 72 hours supernatants were harvested and frozen at −80°C until analysis by ELISA for IL-33 and sST2 as previously described.

### Statistical Analysis

The Kolmogorov-Smirnov test was used to determine whether patient plasma and FF levels of sST2 were normally distributed. sST2 or IL-33 plasma versus FF concentrations were analysed using a paired t-test. Comparison of sST2 levels with varying follicle size or embryo grade were compared by Kruskal-Wallis with Dunn’s post-hoc test or One-Way ANOVA with Bonferroni’s multiple comparison test respectively, using Prism v3 software (GraphPad Software Incorporated, CA, USA).

## Results

### sST2 and IL-33 in Human FF

**Figure 1:**
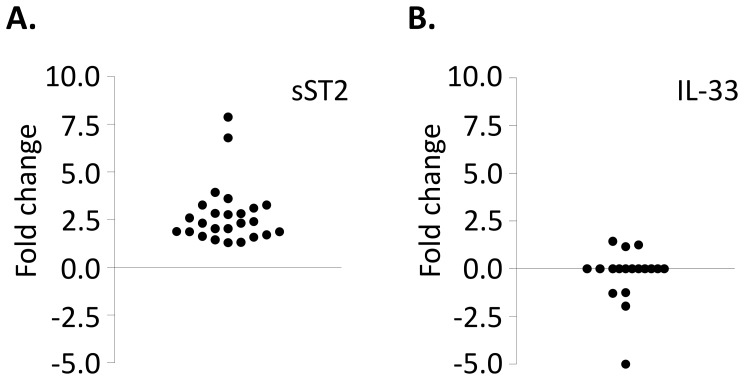
sST2, but not IL-33, is increased in human FFs compared to plasma levels. FFs (at least 5 per patient) and plasma samples were collected from 21 women undergoing ART. Levels of sST2 and IL-33 were determined by ELISA. The mean values of FF levels were compared to the plasma levels, and the fold change in sST2 (A) and IL-33 (B) displayed.

The level of sST2 and IL-33 was measured in the plasma and FF (5–10 per patient) from 21 patients undergoing ART. On the day of oocyte retrieval the mean plasma sST2 concentration was 29.76±2.63 ng/ml and the mean FF sST2 concentration was significantly higher at 76.30±8.42 ng/ml (p<0.0001) (table 1). There was a 2.87-fold enrichment of the mean sST2 in the FF above plasma levels, ranging from 1.30 to 7.90-fold, figure 1A. Individually, FF levels of sST2 were up to 12.07-fold higher than in the peripheral blood plasma. sST2 levels from follicles collected from the same patient were highly variable, for example one patient had FF sST2 levels over the range of 25.97 to 229.58 ng/ml. The maximum sST2 concentration detected in an individual follicle was 269.50 ng/ml, whereas a few follicles (approximately 1%) contained less sST2 than was detectable in the plasma (minimum value 8.41 ng/ml which was four times lower than in the plasma). No correlation was detected between plasma and FF levels of sST2.

Peripheral blood plasma and FF levels of IL-33 were also analysed. IL-33 was detected in the plasma of seven of the twenty-one women analysed, levels were similar in the FF, figure 1B. IL-33 was not detected in the FF of any of the women who did not have detectable IL-33 in the plasma. In patients where IL-33 was detectable FF levels did not correlate with sST2 (data not shown).

Mean plasma or FF sST2 and IL-33 levels do not correlate with the number of follicles nor oocytes retrieved per patient (data not shown). We compared mean FF sST2 and IL-33 levels between women who had a successful or failed IVF/ICSI cycle (clinical pregnancy and live birth –33% of patients), shown in table 1. No difference between the mean levels was detectable between the two groups, and there was no correlation with pregnancy rate and whether a woman had detectable IL-33 or not.

### sST2 and Follicle Size

**Figure 2:**
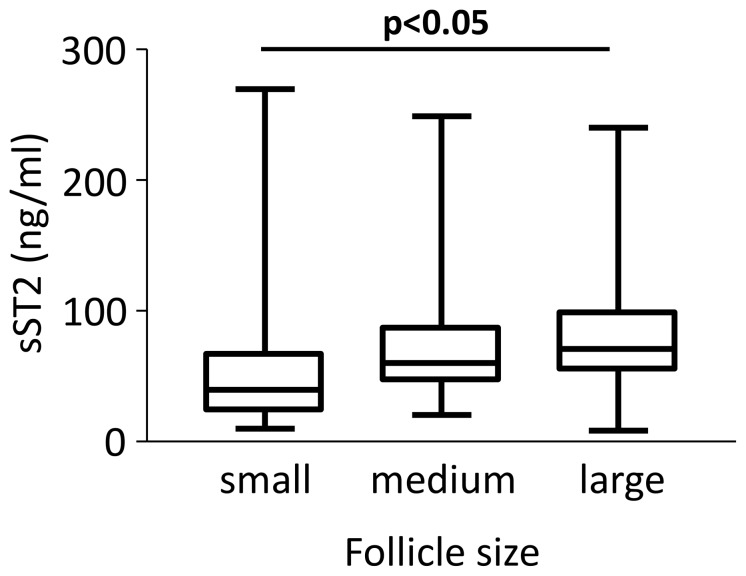
Higher levels of sST2 are expressed in larger follicles. sST2 levels in individual follicles, classified as small (n = 16), medium (n = 35) or large (n = 80), were determined by ELISA. Means ± S.E.M.

sST2 levels were slightly higher in FF from large follicles compared to small follicles (p<0.05), figure 2B. There was no significant difference between sST2 levels when comparing follicles from which oocytes were retrieved than from follicles without successful oocyte retrieval (data not shown).

### ST2 and IL-33 Expression by Granulosa Cells

**Figure 3:**
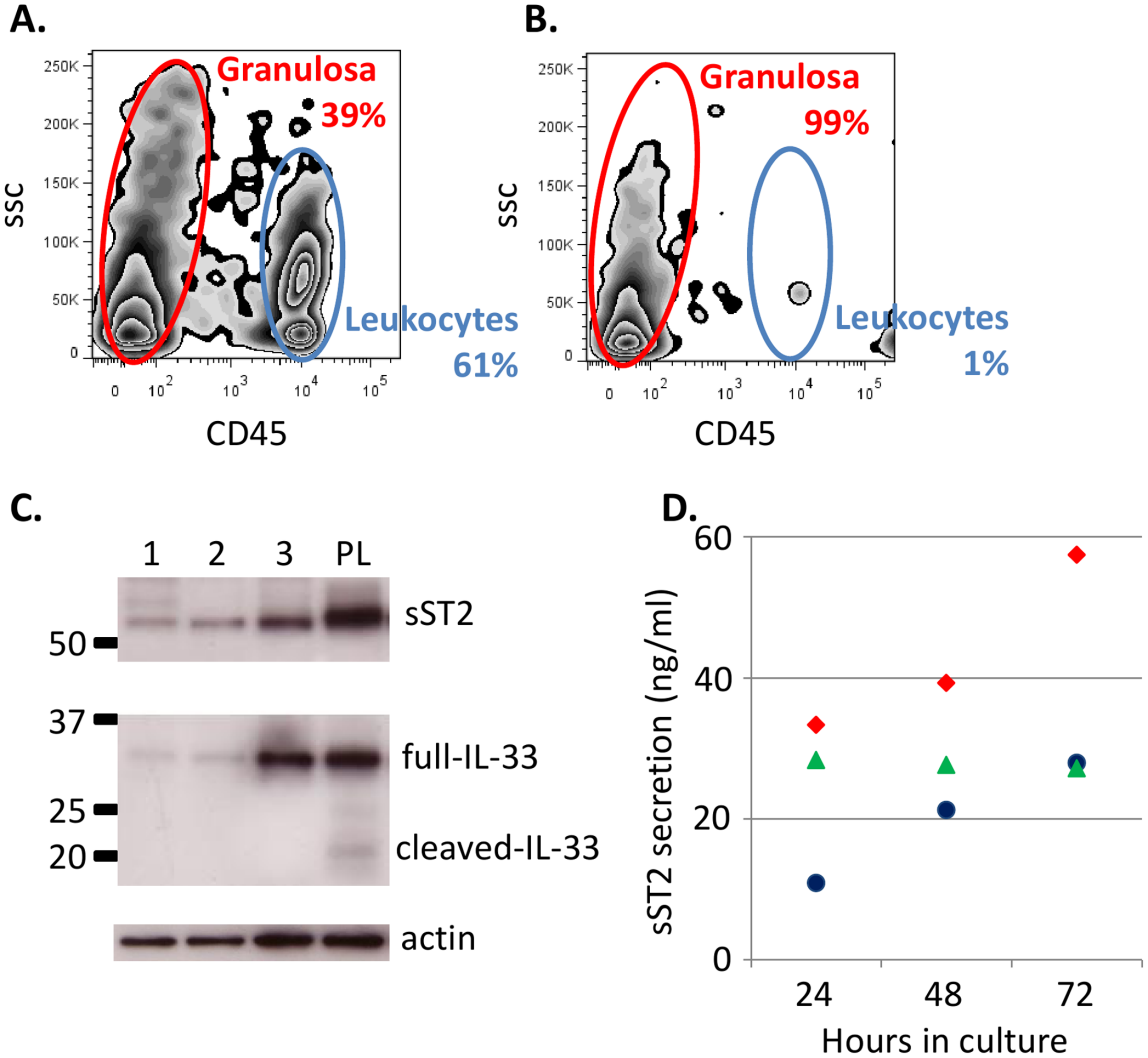
sST2 is secreted by granulosa cells. Aspirated FFs contained a mixture of granulosa cells (CD45 negative) and leukocytes (CD45 positive) (A). Leukocytes were removed by filtration through a 70 µm filter which retained granulosa cells, resulting in >98% purity granulosa cells (B). Full length IL-33 and ST2 in the purified granulosa preparations can be detected by immunoblotting from three patients (1, 2, 3), PL = placenta lysate positive control (C). Purified granulosa secrete sST2 in culture (D), granulosa cell preparations from three patients (red diamonds, green triangles, blue circles) were cultured for up to 72 hours and supernatants assayed by ELISA for sST2. Growth media contained no detectable sST2.

Cells immediately isolated from FFs contained a mixture of CD45 positive leukocyte cells (60–75%) and CD45 negative granulosa cells (25–40%), shown by flow cytometry (figure 3A). Granulosa cell preparations were generated by passing FFs through a 70 µm membrane filter; leukocytes passed through the membrane, whereas the aggregated granulosa cells were retained. The purity of granulosa cell preparations post filtering was assessed as >98% (figure 3B). Immunoblotting of purified granulosa cells from three patients (1, 2, 3) revealed a band of approximately 55 KDa corresponding to sST2, and expression of the full length version of IL-33, but not the smaller cleaved form (figure 3C). A third trimester placenta lysate known to express both ST2 and IL-33 was used as a positive control, and β-actin was detected as a protein loading control (figure 3C).

Purified granulosa cell preparations from three patients were cultured and supernatants harvested at 24, 48 and 72 hours. After 24 hours of culture sST2, but not IL-33, could be detected in the supernatant of all three preparations (figure 3D). sST2 levels were further increased in two of the samples after 48 and 72 hours, and remained constant in one of the cultures.

### Follicular sST2 Levels Compared to Embryo Grade

**Figure 4:**
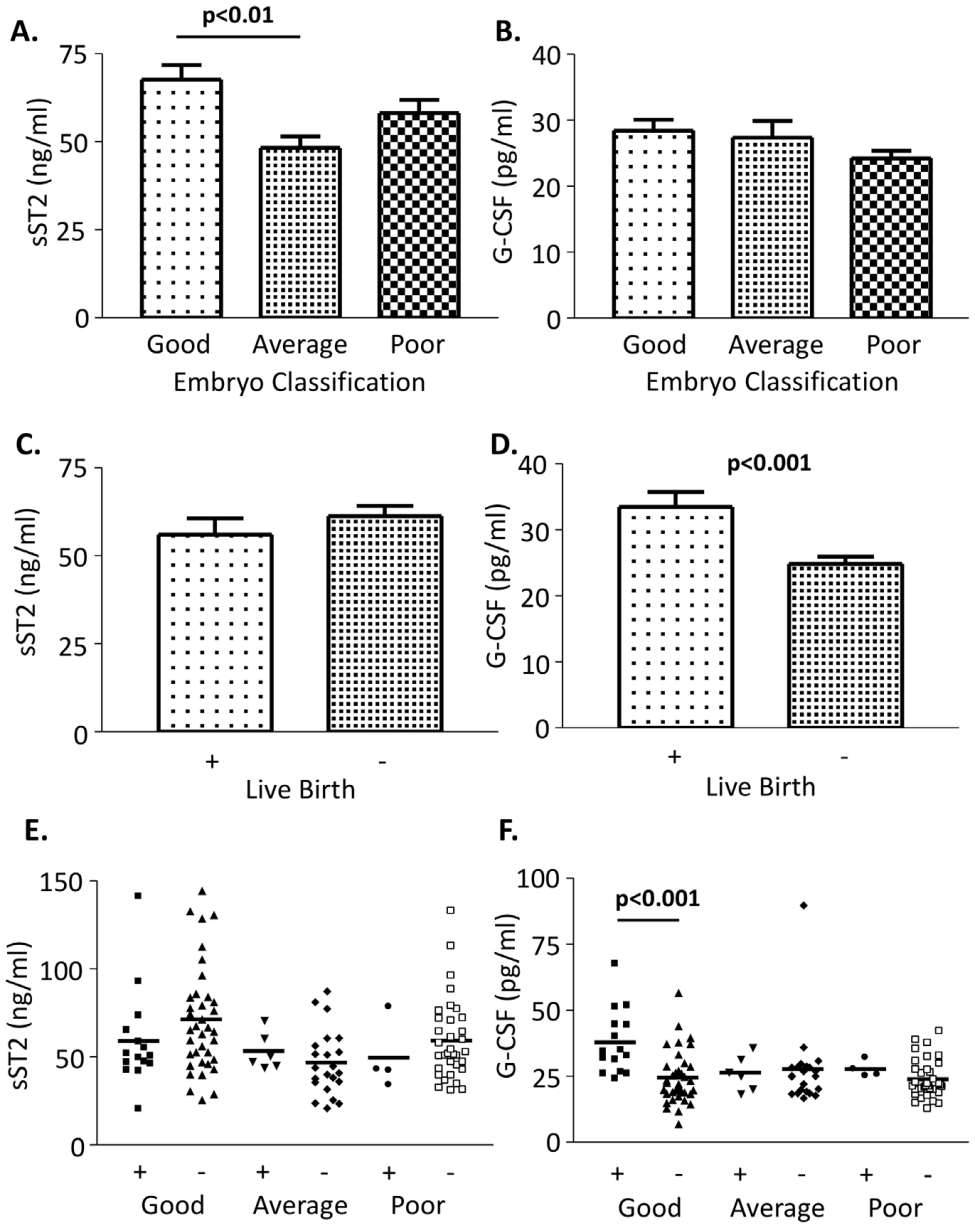
sST2 levels are lower in follicles which produce average quality embryos compared to good quality embryos but levels do not predict IVF success, unlike G-CSF. FFs with known embryo outcome were analysed for levels of sST2 and G-CSF. Embryos were graded good (n = 52), average (n = 28) or poor (n = 38) and cytokine levels compared (A – sST2 and B – G-CSF). Levels of sST2 (C) and G-CSF (D) were compared to live birth outcome, (+; n = 25)) or not (−; n = 88), for all embryo grades, further designated by embryo quality (E and F respectively), good grade/+ (n = 15); good grade/− (n = 37); average grade/+ (n = 6); average grade/− (n = 22); poor grade/+ (n = 4); poor grade/− (n = 34). Bars = mean ± S.E.M (A–D) or scatter plot with bars = mean (E–F).

We next analysed FFs from ICSI cycles where the outcome for the oocyte was known (Liège University samples). Individual FFs were obtained and the oocyte was fertilized by ICSI resulting in embryos (graded good, average or poor quality) which were transferred to the patient. sST2 and G-CSF levels were assessed in the FF (patient information and values given in table 2). FF sST2 levels affected the resulting embryo grade (F = 5.61, p<0.005), sST2 levels were higher where the embryo generated was graded good rather than average (mean 67.65+/−4.13 and 48.23+/−3.25 respectively, p<0.01) (figure 4A). sST2 levels were decreased in poor grade embryos compared to good grade embryos, but this did not reach statistical significance (figure 4A). Plasma or FF sST2 levels did not correlate with plasma FSH, LH or E2 levels (data not shown). G-CSF levels were not different between the groups (figure 4B). As levels of sST2 were different between good and average embryo grades, we next asked if the levels of sST2 in the FF predict pregnancy success. The embryos were returned to the patients, either as a single or double embryo transfer, and the outcome of pregnancy “live birth” or “no pregnancy” was recorded. When two embryos were transferred only “live twin births” or “no pregnancy” were included. sST2 levels did not differ between ICSI cycles that did or did not result in a live birth (figure 4C). G-CSF levels were higher in the follicles of women who had live births (p<0.001; figure 4D and table 2). Next we looked at each embryo grade and compared the live birth rate. G-CSF FF levels were higher in the live birth group, compared to no pregnancy, for the good grade embryos, but not for average or poor grade embryos (figure 4F). For the same samples, FF sST2 levels were lower in the live birth group than no pregnancy group for the good grade embryos, but this did not reach statistical significance (figure 4E). There was no correlation between sST2 and G-CSF levels in the FF (data not shown).

## Discussion

FF is easily obtainable during oocyte collection, therefore factors within the FF which predict pregnancy success have the potential to improve assisted reproductive therapies, particularly in countries where the number of oocytes that can be legally fertilized is limited. In this report we describe high levels of sST2 in human FFs collected at oocyte retrieval in IVF cycles, with higher amounts in larger follicles. Luteinized granulosa cells are one possible source for FF sST2 as we show they express ST2 and secrete sST2 *ex vivo*. ST2 was first identified as a growth response gene [24], and it is possible the higher levels in larger follicles reflect the heightened expansion of these follicles. Alternatively the increased levels may simply be related to increased numbers of granulosa cells in larger follicles. However it is not possible to demonstrate this *ex vivo* as the presence of granulosa cells in extracted FFs is highly variable and not solely dependent on follicle size. Furthermore, it is not possible to determine the numbers of granulosa cells in individual follicles in women undergoing ART.

sST2 levels were further investigated in FF where the associated oocyte was fertilized, embryo grade recorded and transferred back to the patient, and the outcome of the ICSI cycle known. FFs that resulted in good quality embryos contained significantly more sST2 than those leading to average grade embryos. G-CSF levels in these same follicles were also analysed as they have been shown to be a non-invasive biomarker of pregnancy success [25], [26], [27], and like sST2, G-CSF is secreted by human luteinised granulosa cells [28]. As previously reported, FF G-CSF levels do not significantly reflect embryo grade overall [26]. Although slightly lower levels of sST2 were detected in the live birth group (non-significant) FF sST2 levels did not predict cycle outcome. In contrast, as previously reported higher FF G-CSF was present (p<0.01) in the live birth group and this effect was greatest when analysing FFs yielding good quality embryos [4]. sST2 levels cannot therefore be used to improve embryo selection.

Nevertheless, FFs contain very high levels of sST2 of which the role in folliculogenesis is unknown. The best described function of sST2 is as a decoy factor for IL-33, which has dual activity as a transcription factor and cytokine. Its release upon cell death triggers a pro-inflammatory response, and therefore it is also a ‘danger molecule’ or ‘alarmin’ [13]. We could detect IL-33 within granulosa cells, however we did not detect IL-33 within the FF above levels in the circulation, nor was it secreted by luteinized granulosa cells in culture. However sST2 may be present in FF to protect the oocyte from any possible IL-33 release. Several other factors which suppress IL-1 inflammation are highly abundant in FFs, such as apolipoproteins [29], therefore sST2 may be another mechanism to limit IL-1 family driven inflammation in the pre-ovulatory follicle. As levels of sST2 may reflect inflammation, then the observation of slightly higher sST2 in follicles in which the embryos did not result in live birth compared those that led to a live birth, could possibly indicate that there has been an inflammatory reaction in the follicle that has damaged the oocyte, but this has not affected embryo morphology.

Alternative roles for sST2 have been recently described, it can induce cell growth and metastasis [19] and induces monocyte driven inflammation [20]. Therefore sST2 may have an, as yet, undefined role in folliculogenesis.
